# Pattern changes of cutaneous dermatoses among Iraqi women preceding and during the COVID-19 pandemic

**DOI:** 10.12688/f1000research.73719.1

**Published:** 2021-11-17

**Authors:** Galawish A. Abdullah

**Affiliations:** 1 Department of dermatology/ Al- Kindy College of medicine, University of Baghdad, Baghdad, Iraq

**Keywords:** COVID-19, cutaneous dermatosis, female

## Abstract

**Background**: We compared the pattern of cutaneous dermatoses among Iraqi females of all ages between 4 months preceding the coronavirus disease 2019 (COVID-19) pandemic, and the same months 1 year later within the COVID-19 pandemic.

**Methods**: This was a cross-sectional study, that targeted all female patients attending an outpatient clinic for dermatology and venereology in Al-Kindy teaching hospital, Baghdad between October 2019 to the end of January 2020, and the same 4-month duration 1 year later (October 2020 to the end of January 2021) after the COVID-19 peak period had passed and there was no or partial curfew to exclude seasonal impact.

**Results**: A total of 2657 female-patients of all ages were enrolled in this study with 1314 females during the 4 months pre COVID-19, and 1343 females during the pandemic. The mean age of patients presented before the pandemic was 27.2±16.6 years, while the mean age of patients during the pandemic was 28.1±15.6 years with no statistically significant difference in mean ages (P >0.05). Hair loss in general with telogen effluvium specifically increased significantly. Cutaneous contagious viral infections were reduced significantly and specifically of these molluscum contagiosum and condylomata accuminata. Other forms of infections including bacterial and parasitic were also reduced while dermatophytosis was increased but not to a significant level. Acne vulgaris, rosacea, lichen planus, urticaria, pityriasis rosea, seborrheic dermatitis, and vitiligo were increased, but psoriasis, alopecia areata, other types of dermatitis, and melasma were reduced but none to a significant level.

**Conclusions**: The COVID-19 pandemic resulted in changes in the pattern of diseases presented to an out-patient clinic for dermatology and venereology. This could be either related to COVID-19 infection or stress associated with the pandemic, because of curfew, or wearing facemasks which may cause a decrease or increase in certain diseases.

## Introduction

The first reported case of coronavirus disease 2019 (COVID-19) was in Wuhan, China in December 2019 and after that it spread globally. ^1^ Till 16 September 2021 the total confirmed cases all over the world reached 226,236,577 and the total deaths 4,654,548, while in Iraq it reached 1,963,264 and the mortality 21,631 according to the World Health Organization (WHO) reports. ^2^

The Republic of Iraq officially reported the first confirmed case of COVID-19 on February 24, 2020 in Al-Najaf government and after that it started to increase in all cities of Iraq, and many measurements including total and partial curfew on week-ends had been taken in order to limit spread of infection. ^3^ The pattern of diseases including dermatological diseases started changing during the era of COVID-19 pandemic and a lot had been changed since then. ^4^

The aim of this study was to investigate the changes in the profile of dermatological diseases among Iraqi females of all ages before and during the COVID-19 pandemic.

## Methods

### Ethical approval

The study was approved by scientific committee (Research Ethics Committee) of Al-Kindy college of medicine, university of Baghdad. All procedures performed in studies involving human participants were in accordance with the ethical standards of the institutional and/or national research committee and with the 1964 Helsinki declaration and its later amendments or comparable ethical standards (Code: 2019/C081). All patients attending the Al-Kindy teaching hospital in Baghdad are routinely informed that their data could be used for medical research, and their personal information would not be disclosed. In cases below the age of 18 years, the patients care giver would be responsible for giving the approval. If patients do not consent, their data is not shared.

### Study design and setting

This study involved a cross-sectional survey carried out in the out-patient clinic for dermatology and venereology in Al-Kindy teaching hospital, Baghdad. The data was collected from the medical records of all enrolled female patients who attended the out-patient clinic for 8 months, 4 of them before the first COVID-19 case was diagnosed in Iraq (from October 2019 to the end of January 2020), and the other four months 1 year later (from October 2020 to the end of January 2021) to exclude seasonal impact.

### Participants

The data were collected retrospectively from all female patients attending the dermatology out-patient’s clinic during the study period. Inclusion criteria included female patients of any age who were examined by the same dermatologist and had been given a definitive diagnosis. Exclusion criteria was if a definitive diagnosis had not been recorded.

### Variables

The study variables included patients’ age and the definite dermatological diagnosis for each patient.

### Data sources and measurement

Diagnosis was made by clinical examination. Some cases required specific investigation in the form of dermoscopic and wood-light examination, scraping test, routine histopathological examination and immunohistochemistry for selected cases to confirm diagnosis.

### Bias

This study mainly faced two types of bias: selection bias and information bias. Some degree of selection bias was evident because the cases enrolled were examined by the same dermatologist, which was done to ensure that the steps of examination and diagnosis was offered to all patients had similar quality, the other cause of selection bias that this was a single center study. Information bias was limited because the data entry was double checked and that any case without a clear and definite diagnosis was excluded from the study.

### Statistical analysis

The collected data were analyzed by Statistical Package for the Social Sciences ( SPSS), version 22. The descriptive analysis focused on frequencies, and percentages, and percent change. While the Chi-square (goodness of fit) test was used to find the associations between variables and significance of percent change. A P-value ≤ 0.05 was considered statistically significant.

## Results

A total of 2657 female patients were enrolled in this study with 1314 before the COVID-19 pandemic, and 1343 1-year into the pandemic. ^13^ The mean age of patients during the period before COVID-19 was 27.2 ± 16.6 years, while the mean age of patients during the pandemic was 28.1 ± 15.6 years. There was no significant difference between the mean age of the patients before and during the COVID-19 pandemic (P-value > 0.05).

Table 1 shows that hair loss in general was significantly increased during COVID-19 pandemic. Viral infections in general reduced significantly, while diseases like lichen planus, pityriasis rosea, urticaria, rosacea, vitiligo, acne vulgaris, cutaneous fungal infections, and cutaneous leishmaniasis all increased. On the other hand, bacterial and parasitic infections, psoriasis, pruritis, melasma, and dermatitis were decreased but not to a significant level. Uncommon dermatosis included cases with pemphigus vulgaris, bullous pemphigoid, dermatitis herpetiformis, erythema multiforme, and pityriasis rubra pilaris.

**Table 1.** The types of dermatological diseases before and during the coronavirus disease 2019 (COVID-19) pandemic.

Disease diagnosis	Before		During		Percent change	P-value
	N	%	N	%	%	
Hair loss	133	10.1	210	15.6	57.8	**<0.001** *
Viral infection	145	11.0	106	7.9	−26.8	**0.014** *
Fungal infection	88	6.7	97	7.2	10.2	0.508
Bacterial infection	44	3.3	32	2.4	−27.2	0.169
Parasitic infection	66	5.0	49	3.6	−25.7	0.113
Cutaneous leishmaniasis	2	0.2	6	0.4	200	0.157
Acne	203	15.4	222	16.5	9.3	0.357
Rosacea	54	4.1	68	5.1	25.9	0.205
Dermatitis	117	8.9	106	7.9	−9.4	0.461
Psoriasis	72	5.5	61	4.5	−15.2	0.340
Lichen planus	9	0.7	18	1.3	100	0.803
Pityriasis rosea	6	0.5	9	0.7	50	0.439
Urticaria	53	4.0	75	5.6	41.5	0.052
Pruritus	11	0.8	7	0.5	−36.3	0.346
Melasma	51	3.9	40	3.0	−21.5	0.249
Vitiligo	49	3.7	56	4.2	14.2	0.495
Tumor	58	4.4	60	4.5	3.4	0.854
Hirsutism	24	1.8	24	1.8	0	>0.999
Uncommon	129	9.8	97	7.2	−24.8	**0.033** *
Total	1314	100.0	1343	100.0	2.2	0.574

* The Chi-square statistic is significant at the P-value < 0.05 level.

Cutaneous viral infections in general reduced significantly during COVID era, specially molluscum contagiosum and condylomata accuminata, while herpes zoster increased but it was not significant ( Table 2).

**Table 2.** The types of cutaneous viral infection before and during coronavirus disease 2019 (COVID-19) pandemic.

Disease diagnosis	Before		During		Percent change	P-value
	N	%	N	%	%	
Chicken pox	3	2.1	2	1.9	−33.3	0.665
Herpes simplex	4	2.8	4	3.8	0	>0.999
Herpes zoster	6	4.1	9	8.5	50	0.439
Molluscum contagiosum	48	33.1	24	22.6	−50	**0.005** *
Wart (Common wart)	25	17.2	27	25.5	8	782
Wart (Condylomata accuminata)	21	14.5	8	7.5	−61.9	**0.016** *
Wart (Digitat, filiform)	2	1.4	2	1.9	0	>0.999
Wart (Plane wart)	34	23.4	27	25.5	−20.5	0.370
Other (roseola infantum, and hand foot mouth disease)	2	1.4	3	2.8	50.0	0.655
Total	145	100.0	106	100.0	−26.8	**0.014** *

* The Chi-square statistic is significant at the P-value < 0.05 level.

There was a reduction in the percentage of the most common forms of cutaneous bacterial infections after the peak of the COVID-19 pandemic, but it was not statistically significant ( Table 3). Dermatophytosis increased while cutaneous candidiasis decreased but neither were statistically significant ( Table 4).

**Table 3.** The types of cutaneous bacterial infections before and during coronavirus disease 2019 (COVID-19) pandemic.

Disease diagnosis	Before		During		Percent change	P-value
	N	%	N	%	%	
Abscess	5	11.4	4	12.5	−20.0	0.379
Boil	15	34.1	8	25.0	−46.6	0.144
Cellulitis	4	9.1	2	6.3	−50.0	0.414
Eccthyma	2	4.5	1	3.1	−50.0	0.564
Erythrasma	2	4.5	2	6.3	0.0	>0.999
Folliculitis	10	22.7	9	28.1	−10.0	0.819
Impetigo	4	9.1	3	9.4	−25.0	0.705
Others *	2	2.6	3	9.4	50.0	0.665
Total	44	100.0	32	100.0	−27.2	0.169

* Cutaneous tuberculosis, bacillary angiomatosis, and acute paronychia.

**Table 4.** The types of cutaneous fungal infection before and during the coronavirus disease 2019 (COVID-19) pandemic.

Disease diagnosis	Before		During		Percent change	P-value
	N	%	N	%	%	
Candidiasis	22	25.0	13	13.4	−40.9	0.128
Dermatophytosis	66	75.0	84	86.5	27.2	0.142
Total	88	100.0	97	100.0	10.2	0.508

Parasitic infections including scabies and pediculosis decreased during COVID-19 pandemic but also not statistically significant ( Table 5).

**Table 5.** The trend of cutaneous parasitic infection before and during the coronavirus disease 2019 (COVID-19) pandemic.

Disease diagnosis	Before		During		Percent change	P-value
	N	%	N	%	%	
Pediculosis	11	16.6	4	8.2	−63.63	0.071
Scabies	55	83.3	45	91.8	18.18	0.269
Total	66	100.0	49	100.0	−25.75	0.113

Hair loss in general and telogen effluvium specifically increased significantly from the pre COVID-19 period. Cases of female baldness, trichotillomania, and acquired hair shaft anomaly had increased, and cases of alopecia areata and traction alopecia had decreased, however, these changes were not statistically significant ( Table 6).

**Table 6.** Types of hair fall before and during the coronavirus disease 2019 (COVID-19) pandemic.

Disease diagnosis	Before		During		Percent change	P-value
	N	%	N	%	%	
Acquired hair shaft anomaly	5	3.8	7	3.3	40.0	0.564
Alopecia areata	24	18.0	18	8.6	−25.0	0.355
Anagen effluvium	1	0.8	0	0.0	−100.0	–
Female baldness	25	18.8	35	16.7	40.0	0.197
Telogen effluvium	68	51.1	138	65.7	102.9	**<0.001**
Traction alopecia	6	3.5	4	1.9	−33.3	0.527
Trichotillomania	4	3.0	8	3.8	100.0	0.248
Total	133	100.0	210	100.0	57.8	**<0.001**

* The Chi-square statistic is significant at the P-value < 0.05 level.

Seborrheic dermatitis and to little extent contact dermatitis increased, while all other types of dermatitis were reduced, and all are not statistically significant ( Table 7).

**Table 7.** The types of dermatitis types before and after the coronavirus disease 2019 (COVID-19) pandemic.

Disease diagnosis	Before		During		Percent change	P-value
	N	%	N	%	%	
Atopic dermatitis	28	23.9	18	17.0	−35.7	0.140
Contact dermatitis	43	36.8	44	41.5	2.3	0.915
Discoid dermatitis	5	4.3	4	3.8	−20.0	0.739
lichen simplex	10	8.5	6	5.7	−40.0	0.317
Seborrheic dermatitis	10	8.5	18	17.0	80.0	0.131
Xerotic dermatitis	14	12.0	14	13.2	0.0	>0.999
Others **	7	6.0	2	1.9	−71.4	0.096
Total	117	100.0	106	100.0	−9.4	0.461

** Pityriasis alba, juvenile plantar dermatitis, and dyshydrotic dermatitis.

## Discussion

There are many cutaneous manifestations that appeared to be associated with COVID-19 infection. ^5^^,^^6^ To the best of our knowledge, this is the only study investigating the pattern of dermatologic diseases among Iraqi women who presented to an outpatient dermatological clinic in the 4 months before COVID-19 outbreak and compared to the same 4 months one year later that were not in the partial or complete curfew. In this study we choose only female patients of all ages because we believe that females are more aware of their skin and hair than males in our society.

Kutlu and Metin 2020 from Turkey compared 2 months (April and May 2019) to the same months in 2020 which were at the beginning of the era of COVID-19. They found that wart, molluscum contagiosum, and dermatophytosis were significantly decreased while scabies increased over this time period. ^4^ In our study all types of cutaneous infections and infestations which are contagious like viral, bacterial, and parasitic had decreased but not to a significant level except for molluscum contagiosum and condylomata accuminata which may have been due to closure and curfew, decreasing of extra-marital sexual activity, and decreased families visiting each other. Although herpes zoster is considered to be viral infection, it was probably increased as it results from reactivation of a latent virus and not a new infection.

Dermatophytosis in our study had increased 1 year after the start of the pandemic but not to a significant level mostly because many families bought pets to their children during a period of ban, and these were the source of most dermatophytosis in our cases.

The same previous Turkish study found that telogen effluvium and Alopecia areata increased significantly, ^4^ while in our study hair loss in general with telogen effluvium specifically was increased significantly because of fever due to COVID-19 infection which is considered an important cause of telogen effluvium. ^7^ Alopecia areata cases may have decreased because it is a chronic disease and asymptomatic so patients may have postponed visiting a dermatologic clinic to avoid COVID-19 infection.

Lichen planus, pityriasis rosea, and urticaria were increased during COVID-19 era but not to a significant level; the reason may be because pityriasis rosea and urticaria have been reported in many studies to be associated with COVID-19 infection as a direct or indirect cause, ^5^^,^^8^ but lichen planus was not, and because these diseases are itchy and their cutaneous lesions are usually generalized, patients may have worried about their illness and if it is related to COVID-19 infection or not. Psoriasis and melasma are usually chronic diseases, sometimes asymptomatic, so some patients might have postponed attending to dermatology clinic, which may explain the reduction in frequency of these disease during the COVID-19 pandemic; however, Kutlu and Metin reported that psoriasis frequency increased significantly during the COVID-19 pandemic. ^4^

Acne vulgaris and rosacea had increased during the pandemic but also not to a significant level. This increase may have been due to wearing a face mask to reduce the risk of contamination; Han C in 2020 reported an increased flare of acne caused by long time mask wearing during the pandemic, and they attributed that to long- time mask wearing which could increase the flare of acne due to higher temperature and humidity on the surface of facial skin caused by expired air and the perspiration, ^9^ this could also explain the increase in rosacea.

All types of dermatitis decreased except seborrheic dermatitis but not to a significant level. This may be due to the fact that most acne patients have some sort of seborrheic dermatitis, so because acne was increasing, seborrheic dermatitis increased too. Singh M and Abtahi B during COVID-19 (March through April 2020) found that there was an increase of irritant contact dermatitis among the general population likely due to overuse of antiseptic agents and frequent hand and face washing. ^10^^–^^12^

### Limitations of the study

There are some limitations to our study. These are that this research was a single center study and data was only measured over a short period (October to January) which cannot cover all dermatological diseases that could increase or decrease in a certain season.

## Conclusions

The COVID-19 pandemic resulted in changes in the diseases presented to an out-patient clinic for dermatology and venereology. This could be either related to infection with COVID-19 or stress associated with the pandemic or because of closure and wearing mask.

## Data availability

### Underlying data

Figshare: Data_Dr_Galawish.xlsx. https://doi.org/10.6084/m9.figshare.16640476.v1. ^13^

Data are available under the terms of the Creative Commons Attribution 4.0 International license (CC-BY 4.0).

## References

[1] World Health Organization: *Listings of WHO’s response to COVID-19 Online.* World Health Organization;2020 [updated 29 June; cited 2021 10 Aug]. 10.1596/33738 Reference Source

[2] World Health Organization: *WHO Coronavirus (COVID-19) Dashboard.* World Health Organization;2021 [cited 2021 10 Aug]. Reference Source

[3] Novel Coronavirus (COVID-19): Update from Iraq’s Ministry of Health: Government of Iraq:2020 [updated 25 Feb 2020; cited 2021 10 Aug]. Reference Source

[4] Kutlu Ö Metin A : Relative changes in the pattern of diseases presenting in dermatology outpatient clinic in the era of the COVID-19 pandemic. *Dermatol. Ther.* 2020;33(6):e14096. 10.1111/dth.14096 32869938

[5] Genovese G Moltrasio C Berti E : Skin manifestations associated with COVID-19: current knowledge and future perspectives. *Dermatology.* 2021;237:1–12. 10.1159/000512932 33232965 PMC7801998

[6] Galván Casas C Catala A Carretero Hernández G : Classification of the cutaneous manifestations of COVID-19: a rapid prospective nationwide consensus study in Spain with 375 cases. *Br. J. Dermatol.* 2020;183(1):71–77. 10.1111/bjd.19163 32348545 PMC7267236

[7] Olds H Liu J Luk K : Telogen effluvium associated with COVID-19 infection. *Dermatol. Ther.* 2021;34(2):e14761. 10.1111/dth.14761 33405302 PMC7883200

[8] Ehsani AH Nasimi M Bigdelo Z : Pityriasis rosea as a cutaneous manifestation of COVID-19 infection. *J. Eur. Acad. Dermatol. Venereol.* 2020;34:e436–e437. 10.1111/jdv.16579 32359180 PMC7267489

[9] Han C Shi J Chen Y : Increased flare of acne caused by long-time mask wearing during COVID-19 pandemic among general population. *Dermatol. Ther.* 2020;33:e13704. 10.1111/dth.13704 32472634 PMC7300566

[10] Alsaidan MS Abuyassin AH Alsaeed ZH : The Prevalence and Determinants of Hand and Face Dermatitis during COVID-19 Pandemic: A Population-Based Survey. *Dermatol. Res. Pract.* 2020;2020:1–8. 10.1155/2020/6627472 PMC7726962 33376481

[11] Abtahi-Naeini B : Frequent handwashing amidst the COVID-19 outbreak: prevention of hand irritant contact dermatitis and other considerations. *Health Science Reports.* 2020;3(2):e163. 10.1002/hsr2.163 32346616 PMC7185953

[12] Singh M Pawar M Bothra A : Overzealous hand hygiene during the COVID 19 pandemic causing an increased incidence of hand eczema among general population. *J. Am. Acad. Dermatol.* 2020;83(1):e37–e41. 10.1016/j.jaad.2020.04.047 32305441 PMC7161474

[13] Abdullah G : Data_Dr_Galawish.xlsx. figshare. *Dataset.* 2021. 10.6084/m9.figshare.16640476.v1

