# Supplementary material for: Pattern changes of cutaneous dermatoses among Iraqi women preceding and during the COVID-19 pandemic
Source: F1000Res. 2022 Nov 16;10:1165. Originally published 2021 Nov 17. [Version 3] doi: 10.12688/f1000research.73719.3 (PMC9716113; doi:10.12688/f1000research.73719.3)
Supplement: Peer review history

# Peer review: Reviewer response for version 3

**DOI:** 10.5256/f1000research.141156.r155994

**Published:** 2022-12-01

**Authors:** Paola Savoia

**Affiliations:** 1 Department of Health Science, University of Eastern Piedmont, Novara, Italy

In the present version of the paper, the critical issues have been resolved. The work can be accepted for indexing.

Is the work clearly and accurately presented and does it cite the current literature?

Partly

If applicable, is the statistical analysis and its interpretation appropriate?

Partly

Are all the source data underlying the results available to ensure full reproducibility?

Partly

Is the study design appropriate and is the work technically sound?

Partly

Are the conclusions drawn adequately supported by the results?

Partly

Are sufficient details of methods and analysis provided to allow replication by others?

Partly

Reviewer Expertise:

Dermatology

I confirm that I have read this submission and believe that I have an appropriate level of expertise to confirm that it is of an acceptable scientific standard.

# Peer review: Reviewer response for version 2

**DOI:** 10.5256/f1000research.122668.r154783

**Published:** 2022-11-03

**Authors:** Paola Savoia

**Affiliations:** 1 Department of Health Science, University of Eastern Piedmont, Novara, Italy

This paper, entitled “Pattern changes of cutaneous dermatoses among Iraqi women preceding and during the COVID-19 pandemic” aim to compare the pattern of cutaneous dermatoses in this group of people in the period preceding the COVID-19 pandemic, and 1 year later. In my opinion, the subject and the structure of the work are not particularly original, since many papers have been published about it. The interest is mainly linked to the geographic area of the authors.

 The main other criticisms are as follows:

- Since public hygiene measures aimed at preventing COVID-19 are different in different countries and have changed over time, those concerning the area under study should be specified in the introduction.
- The reason why only female patients were included in the study should be specified, and the data possibly compared with those relating to male patients.
- The number of patients included in the two groups is surprisingly similar: the authors should better specify how the two groups were formed. Furthermore, it is not clear whether it is possible that the same patient is placed in both groups, if she had access to dermatological services before and after COVID-19.
- Since among the pathologies that have undergone to a significant reduction there is that of "uncommon dermatoses", and that this includes dermatoses of different origin, it should be stratified, to understand which pathology is actually responsible for the variation.
- Similarly, it is possible to group the pathologies on the basis of the cause (i.e. inflammatory, immunological genesis, etc.). This should in particular replace Table 7, the function of which is unclear.
- The relationship between cases with telogen effluvium and COVID-19 infection should be clarified, specifying how many patients had COVID-19, and the temporal relationship of the infection to hair loss.

Is the work clearly and accurately presented and does it cite the current literature?

Partly

If applicable, is the statistical analysis and its interpretation appropriate?

Partly

Are all the source data underlying the results available to ensure full reproducibility?

Partly

Is the study design appropriate and is the work technically sound?

Partly

Are the conclusions drawn adequately supported by the results?

Partly

Are sufficient details of methods and analysis provided to allow replication by others?

Partly

Reviewer Expertise:

Dermatology

I confirm that I have read this submission and believe that I have an appropriate level of expertise to confirm that it is of an acceptable scientific standard, however I have significant reservations, as outlined above.

# Response

**Published:** 2022-11-07

**Authors:** Galawish Ahmed

Thank you for your time and efforts in reviewing this article, I shall respond to your respected notes as follows:

1. For your note about adding what was the public hygiene measures, I agree that it should be added in the study.
2. Regarding why I chose females as target population, and that was because there were many studies including both genders but my study was to specify changes which occurred in only female because there are many dermatoses noticed more in female than males as hair loss.
3. I did not specify any number of patients to be included in the study, as data were data collected retrospectively for the same months one year apart, because there are certain diseases occurs more with certain season as scabies or leishmaniasis and same patient was excluded if recorded
  before.
4. Uncommon dermatosis was a term used to encompass a very wide range of dermatological disease that were observed in small frequencies (one or two cases), and in the study I mentioned some of these disease like pemphigus vulgaris, bullous pemphigoid, dermatitis herpetiformis, erythema multiforme, and pityriasis rubra pilaris, however the detailed list included more than 80 different dermatoses, so we were not able to stratify them in a more representative way.
5. Regarding grouping of pathologies; First table was showing in general the pathologies as viral, parasitic...etc. and after that each infection was stratified alone to look for in depth changes and details regarding each type of infection, also, dermatitis because there are many types of dermatitis whether exogenous or endogenous, so I clarified type to see if there is increase in contact dermatitis or xerotic dermatitis because of excessive use of alcohol-based sanitizers and detergents or not in our female popular after COVID era. 
6. Regarding number of patients with COVID-19 infection, since the data were taken from the records, we were unable to confirm previous infection with COVID-19, as patients most probably deny getting infected, and also our records are not linked to a central database to confirm COVID-19 infection.

Thank you again for your time and efforts.

# Peer review: Reviewer response for version 2

**DOI:** 10.5256/f1000research.122668.r137326

**Published:** 2022-05-11

**Authors:** Dursun Turkmen

**Affiliations:** 1 Department of Dermatology, Medical Faculty, Inonu University, Malatya, Turkey

The authors made the changes we suggested earlier. We do not have a new proposal. The article can be indexed in this form

Is the work clearly and accurately presented and does it cite the current literature?

Yes

If applicable, is the statistical analysis and its interpretation appropriate?

Yes

Are all the source data underlying the results available to ensure full reproducibility?

Yes

Is the study design appropriate and is the work technically sound?

Yes

Are the conclusions drawn adequately supported by the results?

Yes

Are sufficient details of methods and analysis provided to allow replication by others?

Yes

Reviewer Expertise:

Dermatology

I confirm that I have read this submission and believe that I have an appropriate level of expertise to confirm that it is of an acceptable scientific standard.

# Peer review: Reviewer response for version 1

**DOI:** 10.5256/f1000research.77389.r123154

**Published:** 2022-02-14

**Authors:** Dursun Turkmen

**Affiliations:** 1 Department of Dermatology, Medical Faculty, Inonu University, Malatya, Turkey

The publication is well thought out and well prepared. The COVID-19 pandemic has probably changed the profiles of patients applying to dermatology outpatient clinics all over the world. In the early stages of the pandemic, the applications of patients to outpatient clinics decreased significantly under strict quarantine conditions. Patients avoided applying to hospitals unless there were important reasons. Also, people experienced great anxiety. Due to this concern, our diseases, which are especially affected by psychological conditions, may have increased.

 The article is well written. I think it would be appropriate for publication if the authors broadened the discussion a bit by also using the following articles:

1. Turan C, Metin N, Utlu Z, Öner Ü, Kotan ÖS. Change of the diagnostic distribution in applicants to dermatology after COVID-19 pandemic: What it whispers to us?. ^1^
2. Turkmen D, Altunisik N, Sener S, Colak C. Evaluation of the effects of COVID-19 pandemic on hair diseases through a web-based questionnaire. ^2^
3. Turkmen D, Altunisik N, Mantar I, Durmaz I, Sener S, Colak C. Comparison of patients' diagnoses in a dermatology outpatient clinic during the COVID-19 pandemic period and pre-pandemic period. ^3^

Due to the COVID-19 pandemic, countries have taken various measures. The measures taken and the working conditions of hospitals vary from country to country. To better understand the findings obtained by the authors in their study, we may have the opportunity to make more reliable interpretations in our country, especially when compared to our own studies on the same subject. I am curious about the comments of the authors about the reasons for the differences. At the same time, I think that the article will be more interesting and more understandable with the evaluations to be made.​​​​​​

Is the work clearly and accurately presented and does it cite the current literature?

Yes

If applicable, is the statistical analysis and its interpretation appropriate?

Yes

Are all the source data underlying the results available to ensure full reproducibility?

Yes

Is the study design appropriate and is the work technically sound?

Yes

Are the conclusions drawn adequately supported by the results?

Yes

Are sufficient details of methods and analysis provided to allow replication by others?

Yes

Reviewer Expertise:

Dermatology

I confirm that I have read this submission and believe that I have an appropriate level of expertise to confirm that it is of an acceptable scientific standard, however I have significant reservations, as outlined above.

## References

[1] : Change of the diagnostic distribution in applicants to dermatology after COVID-19 pandemic: What it whispers to us?. *Dermatol Ther* .2020;33(4) : 10.1111/dth.13804 e13804 10.1111/dth.13804 32530503PMC7300472

[2] : Evaluation of the effects of COVID-19 pandemic on hair diseases through a web-based questionnaire. *Dermatol Ther* .2020;33(6) : 10.1111/dth.13923 e13923 10.1111/dth.13923 32594627PMC7361059

[3] : Comparison of patients' diagnoses in a dermatology outpatient clinic during the COVID-19 pandemic period and pre-pandemic period. *Int J Clin Pract* .2021;75(4) : 10.1111/ijcp.13948 e13948 10.1111/ijcp.13948 33332694PMC7883201

# Response

**Published:** 2022-03-20

**Authors:** Galawish Ahmed

Dear Doctor,

 Thank you for reviewing my article, and we appreciate your opinion, I think that only Turkmen et al (2021) is directly related to my article, and I am going to add their results and discuss them in my article.

 Best regards
